# Cattle connection: molecular epidemiology of BVDV outbreaks via rapid nanopore whole-genome sequencing of clinical samples

**DOI:** 10.1186/s12917-021-02945-3

**Published:** 2021-07-12

**Authors:** Jacqueline King, Anne Pohlmann, Kamila Dziadek, Martin Beer, Kerstin Wernike

**Affiliations:** Institute of Diagnostic Virology, Friedrich-Loeffler-Institut, Südufer 10, 17493 Greifswald - Insel Riems, Germany

**Keywords:** BVDV, Tiling PCR, Amplicon sequencing, Nanopore sequencing, Whole-genome sequencing, MinION, BVDV-1, BVDV-2, Cattle

## Abstract

**Background:**

As a global ruminant pathogen, bovine viral diarrhea virus (BVDV) is responsible for the disease Bovine Viral Diarrhea with a variety of clinical presentations and severe economic losses worldwide. Classified within the *Pestivirus* genus, the species *Pestivirus A* and *B* (syn. BVDV-1, BVDV-2) are genetically differentiated into 21 BVDV-1 and four BVDV-2 subtypes. Commonly, the 5’ untranslated region and the N^pro^ protein are utilized for subtyping. However, the genetic variability of BVDV leads to limitations in former studies analyzing genome fragments in comparison to a full-genome evaluation.

**Results:**

To enable rapid and accessible whole-genome sequencing of both BVDV-1 and BVDV-2 strains, nanopore sequencing of twelve representative BVDV samples was performed on amplicons derived through a tiling PCR procedure. Covering a multitude of subtypes (1b, 1d, 1f, 2a, 2c), sample matrices (plasma, EDTA blood and ear notch), viral loads (Cq-values 19–32) and species (cattle and sheep), ten of the twelve samples produced whole genomes, with two low titre samples presenting 96 % genome coverage.

**Conclusions:**

Further phylogenetic analysis of the novel sequences emphasizes the necessity of whole-genome sequencing to identify novel strains and supplement lacking sequence information in public repositories. The proposed amplicon-based sequencing protocol allows rapid, inexpensive and accessible obtainment of complete BVDV genomes.

**Supplementary Information:**

The online version contains supplementary material available at 10.1186/s12917-021-02945-3.

## Background

Bovine viral diarrhea virus (BVDV), a worldwide pathogen of ruminants, belongs to the *Pestivirus* genus of the *Flaviviridae* family [1]. Currently, the four classical *Pestivirus* species, namely *Pestivirus A* (syn. BVDV-1), *Pestivirus B* (BVDV-2), *Pestivirus C* (classical swine fever virus (CSFV)) and *Pestivirus D* (Border disease virus (BDV)), and some atypical pestiviruses are approved by the International Committee on Taxonomy of Viruses (ICTV) [2–4]. The atypical pestiviruses include the HoBi-like pestivirus (species *Pestivirus H*) detected in bovine calves and aborted fetuses [5–7], Bungowannah virus (*Pestivirus F*) identified in pigs in Australia [8], pronghorn antelope pestivirus (*Pestivirus E*) found in blind antelope in the United States [9], giraffe pestivirus (*Pestivirus G*) associated with mucosal-like disease in Kenyan giraffes [10], and novel pestiviruses in other species such as rats [11]. Further putative *Pestivirus* species have been described in various wildlife animals like bats and toothed whales [12, 13], yet await recognition as approved species. The ongoing detection in different host species and wide genomic range emphasizes the great genetic plasticity of the *Pestivirus* genus.

As one of the most important viral pathogens of ruminants across all continents, BVDV infections carry a significant economic and animal welfare impact [14], and have been reported in cattle, sheep, goats, pigs, deer, buffalo, bisons and alpacas [15–21]. While the course of disease ranges from subclinical to acute gastrointestinal, respiratory and reproductive signs, BVDV is also associated with immunosuppression or increasing morbidity rates in affected herds [22, 23]. In addition, intrauterine infection can potentially lead to vertical transmission, resulting in immunotolerant, persistently infected (PI) animals. Due to the lifelong shedding of high amounts of virus by PI animals they are the main source of infection for susceptible animals and uphold the infection cycle [24, 25], which makes them to the main target of disease control programs [26–28].

BVDV-1 and BVDV-2 can be distinguished according to genetic [29] and antigenic [30] characteristics. In Germany, around 95 % of circulating BVDV strains belong to BVDV-1 [29, 31]. Comprising a single-stranded positive-sense RNA genome of approximately 12.3 kb, a sole open reading frame (ORF) is flanked by 5’ and 3’ untranslated regions (5’ UTR and 3’ UTR). The genome encodes one large polyprotein subsequently processed by cellular and viral proteases into the twelve mature proteins N^pro^, C, E^rns^, E1, E2, p7, NS2, NS3, NS4A, NS4B, NS5A, and NS5B [32]. Most phylogenetic analyses compare nucleotide sequences from the 5’ UTR and N^pro^ regions of the viral genome [33, 34], currently resulting in the identification of 21 subtypes of BVDV-1 (1a – 1u) and four subtypes of BVDV-2 (2a – 2d) [35–42]. In comparison, only two subtypes, BVDV-1a and BVDV-1b, were known in the early 1990s [43]. Additionally, point mutations due to the error-prone viral RNA-dependent RNA polymerase and (non-)homologous RNA recombination are well known for pestiviruses, demonstrating the swift genetic evolution and heterogenicity of BVDV [44–48]. Thus, whole-genome sequencing is of great advantage for the precise and systematic genetic evaluation of BVDV. In line, previously conducted molecular epidemiology studies aiding national and international eradication programs are correspondingly built on the foundation of partial 5’ UTR sequences [49–51]. Utilization of whole-genome sequences in combination with epidemiological data would likewise drastically improve molecular epidemiological investigations and offer additional insights of the attained full genome sequences for molecular-epidemiology.

Generation of whole-genome sequences from clinical samples is often challenging due to low virus loads or a limited sample integrity. Today, this can be successfully circumvented by tiling amplicon PCR schemes which have been successfully employed for a range of viruses, including Zika virus, Ebola virus and the recent severe acute respiratory syndrome coronavirus 2 (SARS-CoV-2) [52–55]. In comparison to other target enrichment methods, PCR offers cheap, fast, available and sensitive enrichment and amplification in one step. Amplicon sequencing using the third-generation Oxford Nanopore MinION device has proven to be a popular choice during outbreaks due to real-time, long read, cost effective sequencing with rapid turnaround [56, 57].

Here, we present a combined approach to aid in a rapid, cost effective and accessible generation of whole BVDV genomes from clinical materials, employing a primary tiling RT-PCR covering both BVDV-1 and BVDV-2 species and real-time multiplex nanopore sequencing. Molecular epidemiology analysis of the results provides detailed insights of the circulating BVDV strains and allows the investigation of possible (direct and indirect) connections between outbreaks.

## Results

### Sequencing data

The universal applicability of the protocol was tested on twelve field BVDV samples representing multiple subtypes (BVDV 1b, 1d, 1f, 2a, 2c), with varying viral titres (Cq-values as measured by a panpesti real-time RT-PCR ranging from 19.2 to 32.6), two animal species (11 cattle and one sheep), and a variety of sample matrices (plasma, EDTA blood and ear notch tissue) (Table 1). Of the twelve sequenced samples, ten whole-genome sequences were attained. Sample 1 and sample 2 (both subtype 1b) achieved 96 % full-genome coverage (lack of coverage between nucleotide no. 1376 to 1856) with the proposed protocol while encompassing the highest Cq-values of 28.8 and 32.6, respectively (Additional File 3).

After pooling of the tiled amplicons and purification, all twelve samples had ample nucleic acid concentrations of 200–750 ng/µl, easily meeting the requirements for sequencing. The Ligation Sequencing Kit produced 998,034 demultiplexed, trimmed and quality checked reads in a 6-hour run, while the Rapid Barcoding Kit produced 7,762,073 demultiplexed, trimmed and quality checked reads in a 36-hour run. Exact read count and distribution is documented in Table 1.

**Table 1:** Diagnostic samples included in the study

	Sample ID	Date	State	Species	ST	Matrix	Cq	Read Count		Total Reads	Mapped Reads
								**Ligation**	**Rapid**		
1	2016BVD01435	2016	NI	Cattle	1b	Plasma	28.8	38,635	442,964	481,599	48,145
2	2016BVD01436	2016	NI	Sheep	1b	Plasma	32.6	60,920	89,008	149,928	57,290
3	2018BVD01695	2018	NI	Cattle	1f	Plasma	19.2	66,753	850,149	916,902	824,299
4	2018BVD06212	2018	NW	Cattle	1d	EDTA blood	22.6	89,898	1,255,060	1,344,958	1,277,809
5	2018BVD06214	2018	NW	Cattle	1d	EDTA blood	21.7	88,264	830,138	918,402	870,123
6	2019BVD04871	2019	NW	Cattle	1d	Plasma	24.0	96,192	981,673	1,077,865	1,040,017
7	2019BVD04882	2019	NW	Cattle	1d	Plasma	21.4	118,619	1,226,328	1,344,947	1,278,462
8	2019BVD04888	2019	NW	Cattle	1d	EDTA blood	19.4	83,659	707,704	791,363	427,131
9	2019BVD04889	2019	NW	Cattle	1d	EDTA blood	28.0	157,898	314,612	472,510	229,627
10	2017BVD04597	2017	BW	Cattle	1b	Ear notch	24.8	52,299	272,345	324,644	302,107
11	D66/11–28	2011	TH	Cattle	2a	EDTA blood	24.4	77,725	465,003	542,728	521,038
12	TV02/13, T136	2013	-	Cattle	2c	Plasma	21.5	67,172	327,089	394,261	385,679

Legend: Selected samples with sample information, read counts divided between the Ligation Sequencing Kit and the Rapid Barcoding Kit, and mapped reads. *ST* Subtype, *NI* Lower Saxony, *NW* North Rhine-Westphalia, *BW* Baden-Württemberg, *TH* Thuringia

### Subtype 1b

As one of the most common subtypes in Germany, three BVDV-1b samples were selected for protocol validation. This included two plasma samples from one holding in Lower Saxony (sample 1 and 2, 2016) but affecting different species (cattle and sheep) and both with high Cq-values, and one bovine ear notch sample (sample 10) from Baden-Württemberg (2017) (Table 1).

In the case of sample 1 and sample 2, the question arose whether both animal species kept in the same holding were infected with an identical strain. Due to the high Cq-values of the samples, 96 % whole-genome coverage was achieved (coverage range - sample 1: 0-8761 reads, sample 2: 0-9029 reads; Additional File 1 A) and revealed identity levels of 98.73 % between both samples. Further genetic comparisons showed approximately 91 % identity to the closest whole-genome database relatives: BVDV-1b strains identified in cattle in Brazil, 2014, and China, 2014 (Additional File 3).

**Fig. 1:**
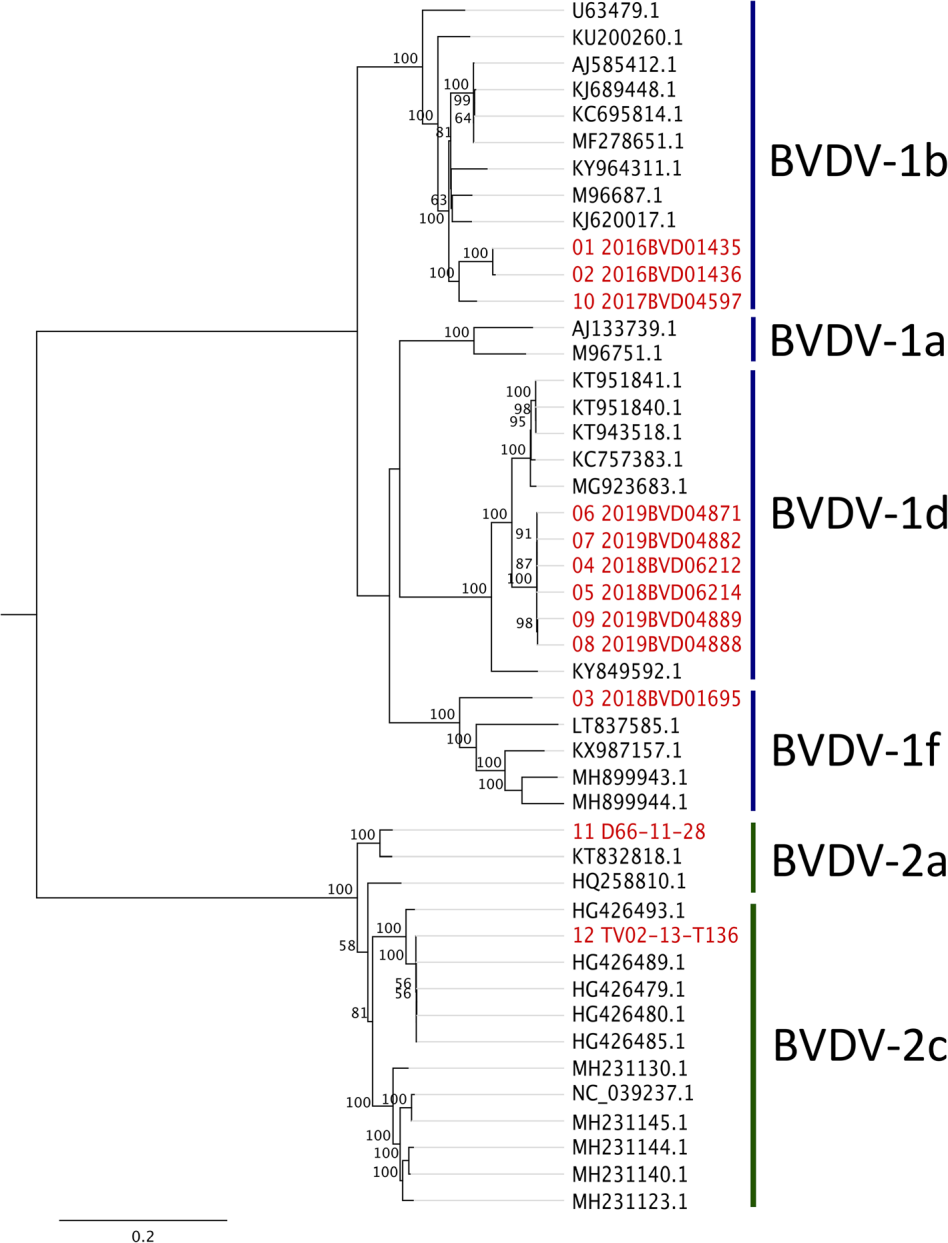
Phylogenetic analysis. Analysis of all novel BVDV samples (highlighted in red) and representative whole-genome sequences from the GenBank database by Maximum Likelihood tree using RAxML with a bootstrap value of 1000 cycles.

In comparison to the previous BVDV-1b samples, sample 10 with a Cq-value of 24.8 was successfully sequenced from ear notch tissue to produce a whole genome with a mean coverage of 15,410-fold (coverage range - sample 10: 12–38,610 reads; Additional File 1 A). Identity levels between the samples 1 and 2 and sample 10 were around 93 %, proving to show greater relatedness than other known available genomes (Fig. 1). Alike the previous BVDV-1b samples, approximately 93 % identity to the described BVDV-1b strains from Brazil and China was recorded as the closest relatives (Additional File 3).

### Subtype 1d

Six BVDV-1d samples collected in North Rhine-Westphalia from 2018/2019 were also fully sequenced (Table 1). Although a frequently identified subtype in Europe, the lack of European BVDV-1d whole-genome sequences leaves strains identified in China (2012) and South Korea (2010) as the most similar available sequences with identity values of approximately 94 %. Mean coverage of the individual mappings ranged from 8455-fold (sample 9) to 64,400-fold (sample 7) (coverage range - sample 4: 15–149,013 reads, sample 5: 10–98,468 reads, sample 6: 11–109,466 reads, sample 7: 12–152,077 reads, sample 8: 10–70,158 reads, sample 9: 11–30,496 reads; Additional File 1 B). Albeit showing low virus loads, indicated by a Cq-value of 28.0, sample 9 was successfully sequenced to produce a whole-genome sequence (Additional File 3).

The six BVDV-1d samples originated from four cattle holdings in close proximity. Samples 4 and 5 originate from one holding (farm 1), as do samples 8 and 9 from a different holding (farm 4). Sample 6 and sample 7 were both collected at again different holdings (farm 2 and farm 3, respectively). Genomic evaluation of the whole-genome sequences allows the establishment of very high identity levels between the individual samples (> 99.6 %) (Table 2). The highest nucleotide identity was observed between sample 4 and sample 5 (99.93 %), taken from the same holding in 2018. Likewise, sample 6 and sample 7 (2019) respectively shared the highest similarity with sample 5, suggesting virus transmission between the farm 1 – farm 2 and farm 1 – farm 3. The identity levels between sample 6 and sample 7 were slightly lower (99.81 %), potentially indicating no direct transmission between farm 2 and farm 3. In comparison, samples 8 and 9, originating from two calves born after one another in the same holding, show lower identity levels with the previously described BVDV-1d samples (approximately 99.7 %), but again share a high sequence similarity of 99.89 % (Fig. 1).

**Table 2:** Full genome identity levels of the consensus sequences of all BVDV-1d samples

Sample ID	04 2018BVD06212	05 2018BVD06214	06 2019BVD04871	07 2019BVD04882	08 2019BVD04888	09 2019BVD04889
04 2018BVD06212	NA	99.93 %	99.83 %	99.88 %	99.74 %	99.75 %
05 2018BVD06214	99.93 %	NA	99.86 %	99.92 %	99.76 %	99.79 %
06 2019BVD04871	99.83 %	99.86 %	NA	99.81 %	99.69 %	99.72 %
07 2019BVD04882	99.88 %	99.92 %	99.81 %	NA	99.71 %	99.74 %
08 2019BVD04888	99.74 %	99.76 %	99.69 %	99.71 %	NA	99.89 %
09 2019BVD04889	99.75 %	99.79 %	99.72 %	99.74 %	99.89 %	NA

Identity is given in %

### Subtype 1f

The sole BVDV-1f sample was successfully sequenced with a 48,083-fold coverage (sample 3) (coverage range - sample 3: 25–132,836 reads; Additional File 1 A). The whole-genome sequence shares only approximately 85 % identity with the nearest related strains from Slovenia (2000) and Italy (2012) (Additional File 3). Continuous circulation of the subtype 1f in the European bovine population is therefore likely. Although still grouping within BVDV-1f, this information confirms the broad genetic range within subtypes and the ongoing evolution over the past decade.

### Subtype 2a and 2c

Both BVDV-2 samples (BVDV-2a, sample 11 and BVDV-2c, sample 12) (Table 1) were successfully sequenced to produce whole genomes with a read coverage of 18,873-fold and 15,874-fold, respectively (coverage range - sample 11: 10–43,367 reads, sample 12: 20–34,672 reads; Additional File 1 C). Identity levels of 90.36 % were established between the described BVDV-2 samples. In comparison to public whole-genome sequences, sample 11 shared the highest identity rate of 96 % with a BVDV-2a strain identified in the United States, 2013/2014 in a PI calf. Unsurprisingly, sample 12 carried high identity levels of 99.9 % with previously sequenced BVDV-2c samples from Germany, 2013, as the sample 12 originated from an animal trial conducted with an isolate collected from the previously sequenced BVDV-2c outbreak in Germany, 2013 (Fig. 1, Additional File 3).

## Discussion

The proposed tiling PCR in combination with real-time nanopore amplicon sequencing successfully produced ten whole genomes and two near-complete genomes of 96 % coverage. Primer sets designed to encompass all known BVDV-1 and BVDV-2 subtypes enabled high viral read coverage, while lowering the necessary sequencing depth, and sequencing of samples with low viral titres. The MinION nanopore device allows a multiplex approach while being portable, accessible and cost-effective, and additionally reduces overall preparation and sequencing time thanks to transposase-based library kits. Alongside successful testing of the presented method, sequencing of five subtypes additionally resulted in twelve new full genome sequences, often devising low identity levels to other available BVDV genomes.

While next-generation sequencers continue to be the gold standard for large data throughput at a moderate cost, clinical application is still limited due to complex protocols and large capital investment [58]. The introduction of cheap, portable and accessible third-generation nanopore devices has started a new era of sequencers. Although the high error rate of MinION reads in comparison to other next-generation sequencers (Illumina, IonTorrent) still exists, ongoing improvements, for example, the R10 flow cells (ONT), and a multitude of bioinformatic analysis tools aid in reduction of the currently standing error profile. Many studies comparing the consequences of next- and third-generation sequencing on the final consensus quality have been conducted, showing that the error rates (93–95 % for ONT, > 99 % for second-generation sequencers) only have a minor impact on the final sequence information and can be well compensated by higher read coverage [59–65]. As is typical for the utilization of a tiling PCR, the coverage of the samples varies depending of multiple factors, including the primer sequence, overlap of multiple primer pairs leading to higher coverage and the quality of the RNA template. The presented data achieved a minimum coverage of at least 10 reads in all areas (except two small dropouts in samples 1 and 2 – Additional File 1). This coverage is sufficient for the production of a reliable consensus sequence.

High-throughput sequencing has improved drastically over the past decade, radically reducing expenditures of time and money, yet in shot-gun approaches the host to virus read ratio still calls for great sequencing depth to achieve whole viral genomes [66]. Especially clinical or field samples often show high and disruptive background read levels. Thus, prior amplification by PCR can aid in reducing costs, sequencing depth and duration. In our study, the used primer pools are designed to cover all subtypes of either BVDV-1 or BVDV-2, additionally eliminating the need for prior subtyping and further reducing expenditures. However, it must be kept in mind that the upstream PCR may introduce biases. As most previous sequencing of 5’ UTR and N^pro^ segments was performed by PCR and Sanger sequencing without the possibility of in-depth analysis, the PCR bias was potentially higher or not identified in earlier studies. In addition, utilization of the upstream PCR could possibly lead to difficulties when identifying novel subtypes. As the primer pools are designed to map more conserved genome regions, acquirement of parts of the genome would still be possible. With the increase of public novel BVDV whole-genome sequences, the primer design could easily be adapted to match all new subtypes.

To date, a mere 378 whole-genome sequences of the entire coding region are available on the NCBI nucleotide database (date: 05.01.2021). In comparison to other RNA viruses, for example influenza A viruses or the new SARS-CoV-2 viruses where databases encode tens of thousands of full genomes [67], the public database comprises only limited BVDV sequence data. This complicates the genomic evaluation and comparison of full-genome sequences, leading to low identity levels between available strains. For example, BVDV-1f sample 3 shared only 85 % sequence identity with the closest available whole-genome sequence although in phylogenetic analysis the sample still clustered within the BVDV-1f group (Fig. 1). Additionally, while the correct allocation only by analysis of short partial 5’ UTR and N^pro^ sequences to the established pestivirus species is usually correct (BVDV-1 or BVDV-2), several publications have indicated limitations of inferring BVDV phylogenies using only partial genes. Especially the 5’ UTR sequence lacks length and diversity, restricting the validity of correct allocations [68–70]. As shown by sample 1 and sample 2, the 5’ UTR region of both sequences are 100 % identical. If only this partial sequence information is considered, both the ovine and the bovine sample would be suspected to be epidemiologically linked. However, full-genome sequencing shows definitive nucleotide differences between the cases with a final nucleotide identity of 98.73 %, thus reducing the likelihood of direct transmission between both animals. Sequencing of BVDV samples would allow greater insights into the genetic diversity and enable the use of molecular epidemiology in outbreaks.

In comparison to samples 1 and 2 (two samples taken simultaneously in the same holding), all six BVDV-1d cases share extremely high similarity rates (> 99.6 %) although sampling was executed over a two-year period (2018–2019) and including four different holdings. The close genetic relations could be attributed to a genetic stabilization of the circulating BVDV-1d strain in PI animals that are immunotolerant to this particular strain. Full-genome sequencing can allow in-depth molecular epidemiological analysis of the viruses in the affected holdings, thus enabling the identification of potential transmission events between individual cases. Here, the highest identity levels were observed between samples of one holding (samples 4 and 5). However, even though sample 6 and sample 7 were collected in the following year in two different holdings, both genomes were highly similar to especially sample 5. This indicates direct transmission of the determined BVDV-1d strain from farm 1 to farm 2 and 3, potentially by mechanical, human or other vectors. As the nucleotide identity of sample 6 and sample 7 is lower, transmission between the individual holdings is less likely than transmission from farm 1 to farm 2 and farm 3 individually. Unsurprisingly, samples 8 and 9 share high identity levels. Although samples 8 and 9 show slightly lower nucleotide similarity to the other BVDV-1d cases, a transmission event from the originating holding is still likely. Due to the longer time period and thus evolution potential of the strain alongside near eradication of BVDV in Germany resulting in less potential infection sources, a close relation of all BVDV-1d samples is highly probable. Additional epidemiological outbreak investigation data could aid in the detection of certain transmission routes.

BVDV strains show a broad identity range within and between the individual subtypes. For example, BVDV-2a sample 11 and BVDV-2c sample 12 carried a nucleotide sequence divergence of approximately 10 %, while BVDV-1f sample 3 showed divergences of around 15 % within the respective subtype. Especially BVDV-1 subtypes have been reported with up to 16 % divergence within the same group and up to 23 % divergence between the subtypes [29, 71]. Due to the lack of specific criteria for subtyping of new virus isolates, an underlying uncertainty exists in the BVDV classification system. A clear and encompassing approach with standardized taxonomy would greatly aid the analysis of genomic BVDV data [29].

## Conclusions

With the help of the described and validated protocol, future rapid, accessible and cost-effective whole-genome sequencing of prior and circulating BVDV strains is facilitated. The ongoing growth and never-ending identification of novel subtypes calls for precise genetic evaluation, especially in areas utilizing BVDV vaccinations to ensure functionality of the vaccines. Phylogenetic and network analysis would likewise considerably profit from the production of novel BVDV whole genomes, allowing molecular epidemiological tracing of outbreaks and aiding in eradication programs.

## Methods

### Sample selection and RNA extraction

A panel of twelve BVDV samples were selected after subtyping based on the 5’ UTR sequences as previously described [72] to include both BVDV species and common subtypes 1b (*n* = 3), 1d (*n* = 6), 1f (*n* = 1), 2a (*n* = 1) and 2c (*n* = 1). All BVDV-1 samples were collected as field samples in Germany between 2016 and 2019. The samples 1 (cattle) and 2 (sheep) originated from the same holding. BVDV-2 samples were collected in 2011 (subtype 2a, *n* = 1, field sample) and 2013 (subtype 2c, *n* = 1, sample collected during an experimental infection study at the Friedrich-Loeffler-Institut, Germany). Plasma, EDTA blood or ear notch material was the chosen sample matrix. With Cq-values ranging from 19.2 to 32.6, including multiple sample matrices and two host species (cattle and sheep), a respective subset mirroring field conditions was chosen for full-genome sequencing. Additional sample information can be found in Table 1. RNA of the respective strains was extracted from plasma and EDTA blood samples with the QIAamp Viral RNA Mini Kit (Qiagen, Hilden, Germany) and from the ear notch with the RNeasy Mini Kit (Qiagen, Hilden, Germany) according to the manufacturer’s instructions.

### Tiling PCR and purification

**Fig. 2:**
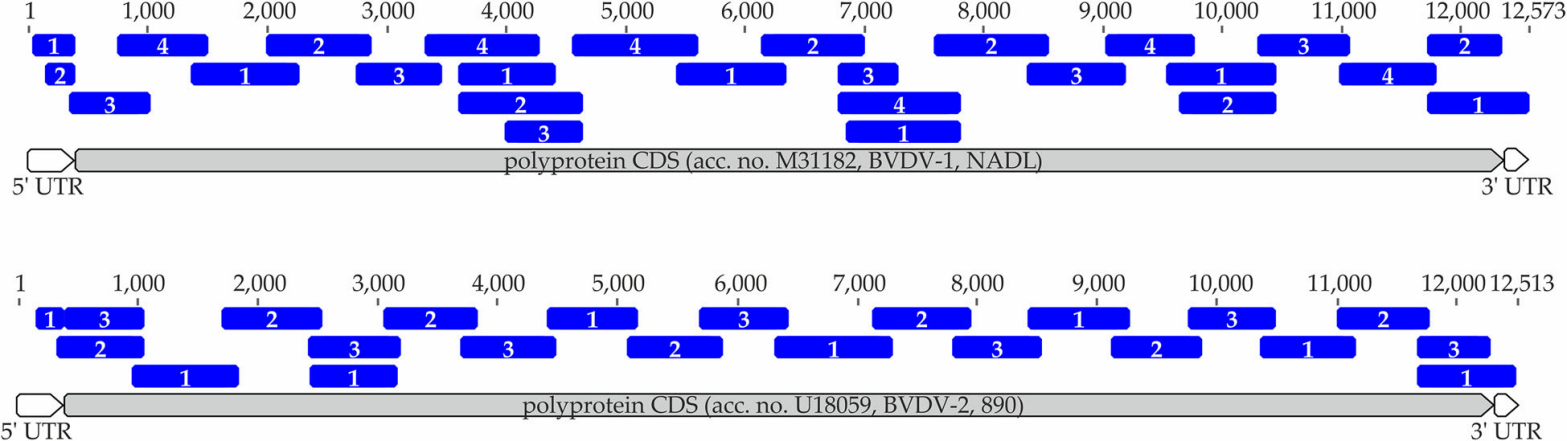
Primer design, coverage and pooling guide for BVDV-1 and BVDV-2 strains. Pooling was performed in accordance with the numbers of the tiles (1-4), resulting in four primer pools for BVDV-1 (upper panel) and three primer pools for BVDV-2 (lower panel).

To facilitate full-genome sequencing, selected BVDV samples were amplified by tiling PCR. Primers were designed conferring to the sample genotypes and carefully selected to cover all subtypes of a respective BVDV species. To minimize the necessary number of PCR reactions while still guaranteeing functionality (for example avoiding overlapping primer pairs which would result in too small PCR fragments) the primers were pooled in equal parts (20pmol/reaction for each primer). This resulted in four primer pools for BVDV-1 samples and three primer pools for BVDV-2 samples. All primers were designed to cover approximately 900 bp tiles with overlap to ensure full coverage (Fig. 2). Exact primer sequences and pooling instructions are recorded in Additional File 2.

The RT-PCR was performed using the SuperScript III One-Step RT-PCR kit (ThermoFisher Scientific, Darmstadt, Germany). For amplification, the reaction was carried out at the following temperature profile: reverse transcription at 50 °C for 30 min, PCR initiation at 94 °C for 2 min, and 40 cycles of a three-step cycling consisting of the denaturation at 94 °C for 15 s, annealing at 56 °C for 30 s, and extension at 68 °C for 90 s. Ensuing pooling of the respective PCR products (BVDV-1–4 pools/sample, BVDV-2–3 pools/sample; Fig. 2), a subsequent purification step with the AMPure XP Magnetic Beads (Beckman Coulter, Fullerton, US) was performed in a 1:1 sample to beads ratio. Final quantification was achieved with the NanoDrop 1000 Spectrophotometer (ThemoFisher Scientific, Waltham, US).

### Whole-genome MinION sequencing

The purified BVDV PCR products were sequenced on a MinION Mk1C platform (Oxford Nanopore Technologies - ONT, Oxford, UK). Two multiplex sequencing methods were tested. This includes a transposase-based approach with the Rapid Barcoding Kit (SQK-RBK004, ONT) for rapid library preparation, and an end-ligation approach with the Ligation Sequencing Kit (SQK-LSK109, ONT) in combination with the Native Barcoding Expansion (EXP-NBD104, ONT) for greater throughput.

While the Rapid Barcoding Kit utilizes a two-step transposase-directed method for simultaneous DNA cleaving and attachment of twelve distinct barcodes to the cleaved ends, followed by pooling of the multiplexed samples in the desired ratio and addition of sequencing adapters (approximately 20 min library preparation), the Ligation Sequencing Kit first repairs the DNA ends and uses dA-tailing for the ligation of dT-tailed unique barcodes, followed by pooling of the multiplexed samples and subsequent cohesion of sequencing adapters onto the “sticky” ends of the barcodes (approximately 3 h library preparation). Both pooled and multiplexed libraries containing all 12 respective samples underwent real-time sequencing on a Mk1C device with a standard R9.4.1 flow cell (FLO-MIN106D, ONT) in a 6-hour run (Ligation Sequencing Kit)/36-hour run (Rapid Barcoding Kit) utilizing fast basecalling with the basecaller Guppy (v3.2.9, ONT). The attained demultiplexed, quality checked and trimmed FastQ files were employed for further analysis.

### Data analysis and availability

Analysis of the obtained sequencing data was conducted in Geneious Prime (v2021.0.3, Biomatters, New Zealand). After a further quality trimming step to remove all primer sequences and short reads (< 50 bp reads), full-genome sequences were produced in an iterative map to reference approach with MiniMap2 [73] and the respective highest quality consensus sequence (threshold 60 % identity) was selected for further phylogenetic analysis. Nucleotide alignment of the novel sequences and a representative subset of available BVDV full genome sequences in the INSDC database was conducted with MAFFT [74] as the foundation of the maximum likelihood analysis using RAxML [75] including 1000 bootstrap replicates. Additional sequence identity analyses were performed using NCBI BLAST (BLAST: Basic Local Alignment Search Tool) online.

All respective full-genome sequences obtained in this study were uploaded to the INSDC under the accession numbers MW528224 to MW528235 (Additional File 3).

## Supplementary Information

**Additional file 1.** Mapping coverage presented in a logarithmic scale.

**Additional file 2.** Primer sequences and pooling guide.

**Additional file 3.** Additional sequencing data and GenBank accession number.

## Data Availability

The data presented in this study are openly available in the INSDC repository under the accession numbers MW528224 to MW528235.

## Abbreviations

BLAST: Basic Local Alignment Search Tool
BDV: Border disease virus
BVDV: Bovine viral diarrhea virus
BW: Baden-Württemberg
CSFV: Classical swine fever virus
Cq: Quantification cycle
EDTA: Ethylenediaminetetraacetic acid
ICTV: International Committee on Taxonomy of Viruses
INSDC: International Nucleotide Sequence Database Collaboration
NCBI: National Center for Biotechnology Information
NI: Lower Saxony
NW: North Rhine-Westphalia
ONT: Oxford Nanopore Technologies
ORF: Open reading frame
PI: Persistently infected
RAxML: Randomized Axelerated Maximum Likelihood
RNA: Ribonucleic acid
RT-PCR: Reverse transcription – Polymerase chain reaction
SARS-CoV-2: Severe acute respiratory syndrome coronavirus 2
ST: Subtype
TH: Thuringia
UTR: Untranslated region
