# Supplementary material for: Cattle connection: molecular epidemiology of BVDV outbreaks via rapid nanopore whole-genome sequencing of clinical samples
Source: BMC Vet Res. 2021 Jul 12;17:242. doi: 10.1186/s12917-021-02945-3 (PMC8272987; doi:10.1186/s12917-021-02945-3)
Supplement: Supplementary file 1 — Additional file 1. Mapping coverage presented in a logarithmic scale. [file 12917_2021_2945_MOESM1_ESM.docx]

**Cattle Connection: Molecular Epidemiology of BVDV Outbreaks via Rapid Nanopore Whole-Genome Sequencing of Clinical Samples**

Jacqueline King, Anne Pohlmann, Kamila Dziadek, Martin Beer and Kerstin Wernike

**Legends:**

**Additional file 1A:** Mapping coverage presented in a logarithmic scale of all BVDV-1b and BVDV-1f samples (sample number 1, 2, 3 and 10)

**Additional file 1B:** Mapping coverage presented in a logarithmic scale of all BVDV-1d samples (sample number 4 ‑ 9)

**Additional file 1C:** Mapping coverage presented in a logarithmic scale of all BVDV-2a and BVDV-2c samples (sample number 11 and 12)

**Additional file 1**

A: Mapping coverage presented in a logarithmic scale of all BVDV-1b and BVDV-1f samples (sample number 1, 2, 3 and 10)


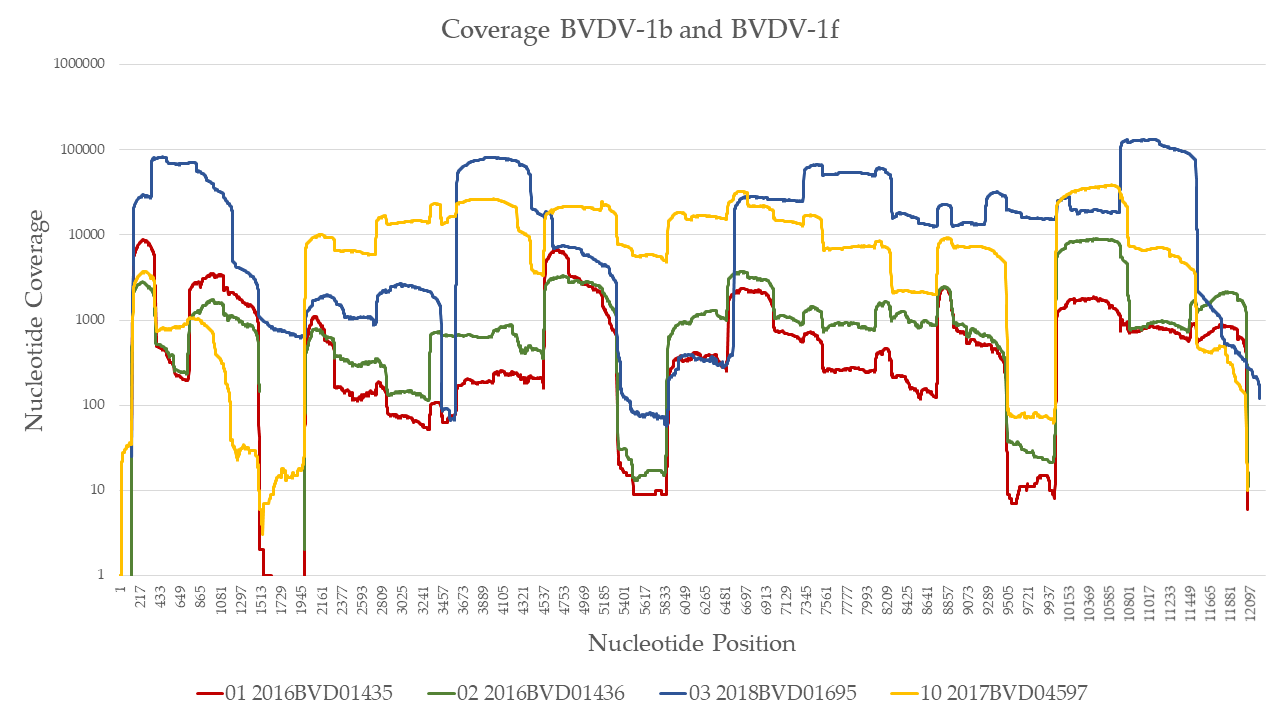


B: Mapping coverage presented in a logarithmic scale of all BVDV-1d samples (sample number 4 – 9)


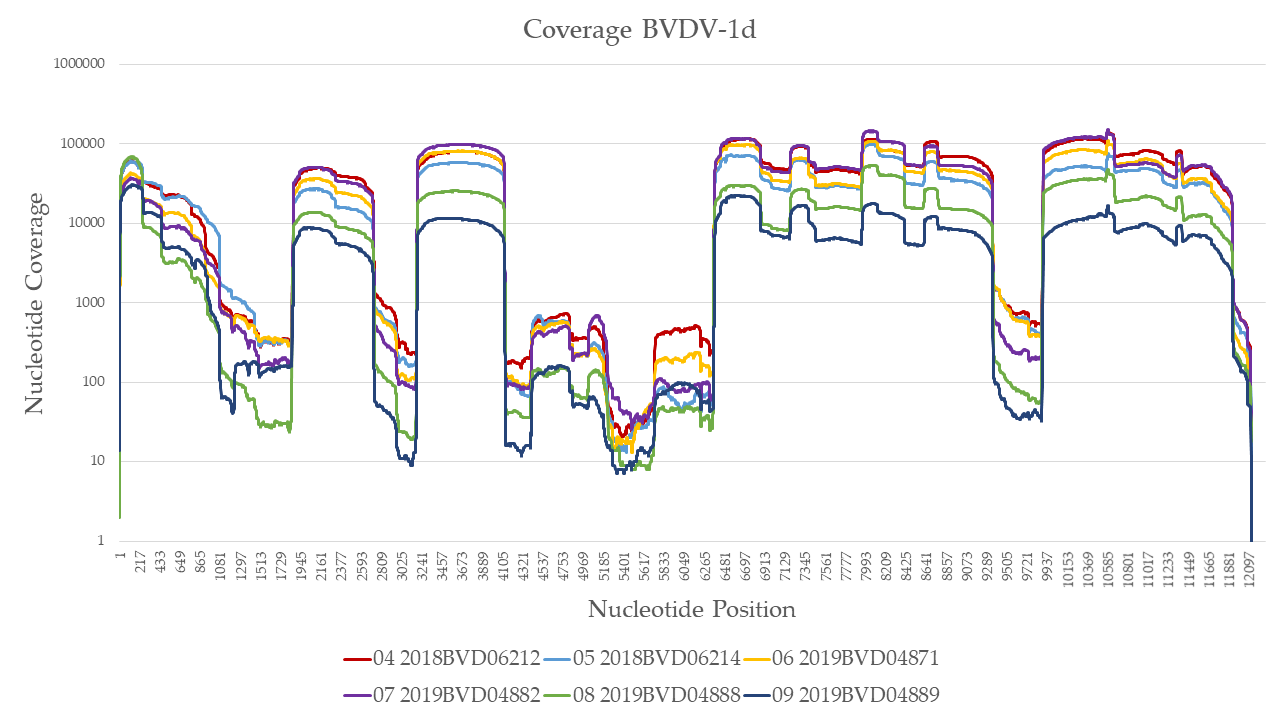


C: Mapping coverage presented in a logarithmic scale of all BVDV-2a and BVDV-2c samples (sample number 11 and 12)

**
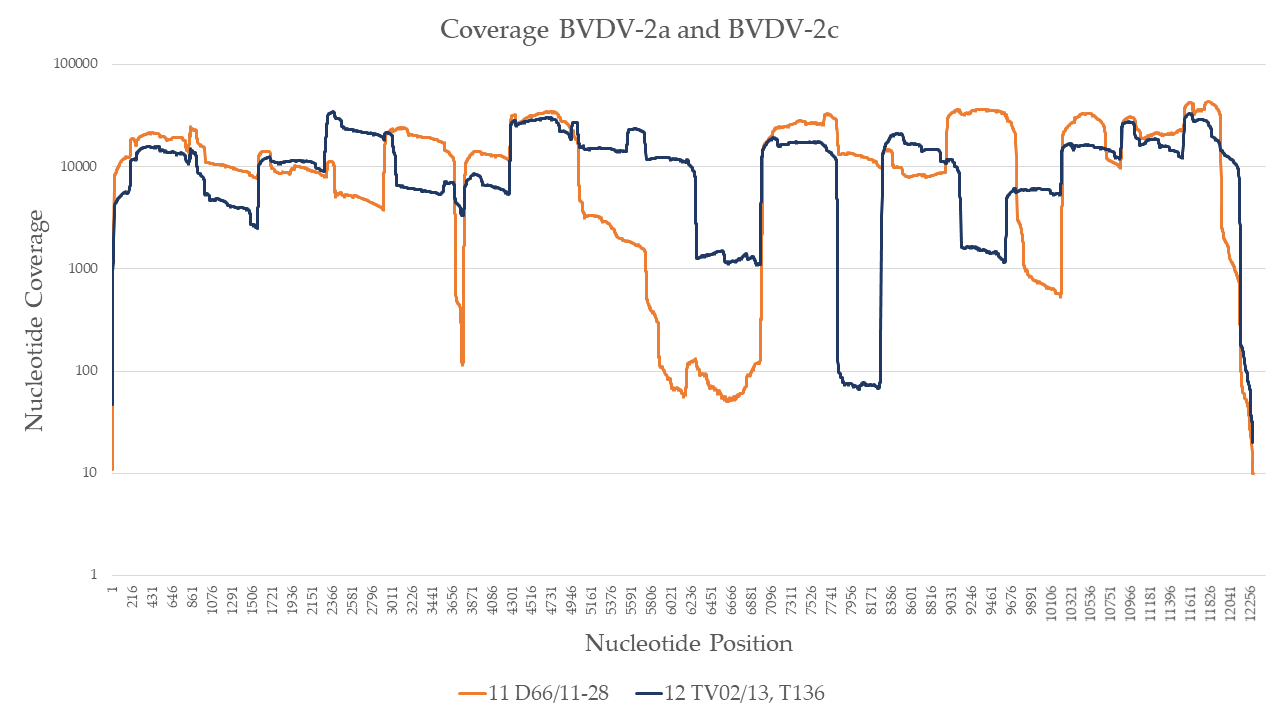
**
