# Supplementary material for: Cattle connection: molecular epidemiology of BVDV outbreaks via rapid nanopore whole-genome sequencing of clinical samples
Source: BMC Vet Res. 2021 Jul 12;17:242. doi: 10.1186/s12917-021-02945-3 (PMC8272987; doi:10.1186/s12917-021-02945-3)
Supplement: Supplementary file 2 — Additional file 2. Primer sequences and pooling guide. [file 12917_2021_2945_MOESM2_ESM.docx]

**Cattle Connection: Molecular Epidemiology of BVDV Outbreaks via Rapid Nanopore Whole-Genome Sequencing of Clinical Samples**

Jacqueline King, Anne Pohlmann, Kamila Dziadek, Martin Beer and Kerstin Wernike

**Legends:**

**Additional file 2A:** Primer sequences and pooling guide. Pooling results in four primer pools for BVDV-1. Pooling is conducted according to the table with equal parts of each primer in every pool. Primer starting position is numbered according to the reference strain NADL (Acc. No. M31182 – NCBI Database).

**Additional file 2B:** Primer sequences and pooling guide. Pooling results in three primer pools for BVDV-2. Pooling is conducted according to the table with equal parts of each primer in every pool. Primer starting position is numbered according to the reference strain 890 (Acc. No. U18059 – NCBI Database).

**Additional file 2**

A: Primer sequences and pooling guide. Pooling results in four primer pools for BVDV-1. Pooling is conducted according to the table with equal parts of each primer in every pool. Primer starting position is numbered according to the reference strain NADL (Acc. No. M31182 – NCBI Database).

| **Pool** | **Primer Name** | **Sequence** | **Primer Starting Position (nt)** |
| --- | --- | --- | --- |
| **Pool 1** | 5’cp7 | CTCGTATACATATTGGACACTC | 22 |
|  | UTR51 | CAA CTC CAT GTG CCA TGT AC | 375 |
|  | BVDV1_2473F | GGT ATG ATG GAT GCR AGY G | 1361 |
|  | BVDV1_3383R | TCA TAR GGC CAC CAA TCT GG | 2252 |
|  | BVDV1_4757F | GAA RTR GTG ATG ATG GGC AAC | 3602 |
|  | BVDV1_5767R | CGC CYT CTC CAC TYT CAT C | 4400 |
|  | BVDV1_6868F | GGG CCT GCC GTR TGY AAG | 5432 |
|  | BVDV1_7787R | TTT GGY TGW GGC ATY TGG CA | 6330 |
|  | BVDV1_8284F | TGT GAR AAG AGG GTG AGG GT | 6839 |
|  | BVDV1_12505R | GGA CRT ACC CYT TWG CAG C | 7788 |
|  | BVDV1_9524F | TCA GAG GTC GGG ATC ACA ATA | 9524 |
|  | BVDV1_15156R | CYT TGG TTT CTT YTC TTC TTC TTC | 10430 |
|  | BVDV1_16426F | AAC ACC AGY AGT TAC ATG GC | 11717 |
|  | BVDV-1-3UTR-R | GGC TGT TAA GGG TCT TCC C | 12557 |
| **Pool 2** | BVDV1_F | GGT AGC AAC AGT GGT GAG TTC | 139 |
|  | UTR51 | CAA CTC CAT GTG CCA TGT AC | 375 |
|  | BVDV1_3100F | GAC ACC AAT GCW GAR GAT GG | 1988 |
|  | BVDV1_3987R | GTC CCW GTC CAT CCT ATR G | 2853 |
|  | BVDV1_4757F | GAA RTR GTG ATG ATG GGC AAC | 3602 |
|  | BVDV1_5852R | GAC CAG TTY ARC TCT ATR AGT G | 4626 |
|  | BVDV1_7578F | AAA GCA GTK ATA GAG GAG ATA GG | 6134 |
|  | BVDV1_8456R | TAG TGG TAG TCC TTT GAC CC | 6992 |
|  | BVDV1_9031F | CTA GAR GAC ACM ACC CAC CT | 7586 |
|  | BVDV1_13241R | CCA ATT GTC TGR ACT GCT TCA | 8521 |
|  | BVDV1_16426F | AAC ACC AGY AGT TAC ATG GC | 11717 |
|  | BVDV1_17049R | CCG ACA GCY GCC ATR AGC | 12322 |
|  | BVDV1_14333F | AAA GAT YGG RTT GGA TGA AGG | 9631 |
|  | BVDV1_15156R | CYT TGG TTT CTT YTC TTC TTC TTC | 10430 |
| **Pool 3** | BVDV1_1260F | TGC TGC AGA GGC CCA CTG | 334 |
|  | BVDV1_2132R | ACC ACT ATC GTR GCA TCY GG | 1007 |
|  | BVDV1_3860F | ATG GAW GAC AAC TTT GAA TTY GG | 2774 |
|  | BVDV1_4576R | AYC TCY AGG TCA AAC CAR TAT TG | 3434 |
|  | BVDV1_5113F | CTG ATG RTT AGY TAT GTG ACA GA | 3992 |
|  | BVDV1_5852R | GAC CAG TTY ARC TCT ATR AGT G | 4626 |
|  | BVDV1_8364F | ACC AAT GCC ATY GAR TCA GG | 6773 |
|  | BVDV1_8878R | CYT CYC CAT TCC TTA TTT TTG G | 7265 |
|  | BVDV1_13054F | ATA TCY GTS ATG CTG GGG G | 8354 |
|  | BVDV1_13889R | AYC CRA CCC CRG TAT ATC TC | 9169 |
|  | BVDV1_14992F | ARG GGC ACA TGG CAT CAG C | 10290 |
|  | BVDV1_15780R | TGT CTT RGC TTC RGG GTA TTG | 11054 |
| **Pool 4** | BVDV1_1762F | GGG AGA GTA ACT GGY AGT GA | 740 |
|  | BVDV1_2618R | CCC TCA GTA AGR TTG GCT TG | 1487 |
|  | BVDV1_4441F | AGG GGC CWG TRG AAA AGA C | 3321 |
|  | BVDV1_5396R | GRG TYA RGA AGT CGG CCC A | 4256 |
|  | BVDV1_5749F | CTG GAC TTT ATG TAC TAC ATG CA | 4544 |
|  | BVDV1_7052R | CCT TGG TGY GTR TAR GCC C | 5589 |
|  | BVDV1_8364F | ACC AAT GCC ATY GAR TCA GG | 6773 |
|  | BVDV1_12505R | GGA CRT ACC CYT TWG CAG C | 7787 |
|  | BVDV1_13718F | CTC ARA GAG ATA AAA CCA GTK GC | 9017 |
|  | BVDV1_14473R | GTY TTW GCT CTA TTT GAC ATG G | 9750 |
|  | BVDV1_15679F | ATA CCA AAA AAT GAG AAR AGA GAT G | 10973 |
|  | BVDV1_16511R | CYC TYT CTC CRC TTG AAT CC | 11782 |

B: Primer sequences and pooling guide. Pooling results in three primer pools for BVDV-2. Pooling is conducted according to the table with equal parts of each primer in every pool. Primer starting position is numbered according to the reference strain 890 (Acc. No. U18059 – NCBI Database).

| **Pool Nr.** | **Primer** | **Primer Sequence** | **Primer Starting Position (nt)** |
| --- | --- | --- | --- |
| **Pool 1** | BVDV2_F | AGC GGT AGC AGT GAG TTC ATT | 142 |
|  | UTR51 | CAA CTC CAT GTG CCA TGT AC | 375 |
|  | BVDV2_967F | AAG ATG AAA ATA GCC CCM AAA GA | 965 |
|  | BVDV2_1859R | TTA TTC TCC AAC TTC TTA CCA AGG | 1825 |
|  | BVDV2_2449F | ATG CTA ATA ACA GGR GCA CAG | 2438 |
|  | BVDV2_3177R | TAC ATA CCT RTA CCC ACT CTC | 3146 |
|  | BVDV2_4648F | AYA TTG ACK TAG CAG GCT TCC | 4416 |
|  | BVDV2_5403R | TCY TCA AAG TCT GCG AGT GTC | 5149 |
|  | BVDV2_6912F | GGT AGA GTA TTC ATA TAT ATT CTT AGA | 6310 |
|  | BVDV2_7897R | CGT CCT CTC CTA GTT TTC TTG C | 7274 |
|  | BVDV2_9022F | GCA ACA GAY GAG YTA GTC AAA G | 8420 |
|  | BVDV2_9882R | GTT AGA GAT TGT TAG GTC ATA TGT G | 9256 |
|  | BVDV2_10957F | GAG GCY TAC CTT AAA CTC AAA G | 10335 |
|  | BVDV2_11765R | GGG TCC TGG AAT GAA TCC C | 11145 |
|  | BVDV2_12271F | AAC ACC AGT AGT TAC ATG GCA G | 11672 |
|  | BVDV2-3UTR-R | CCT CTA GTC CAA CCA TGG AC | 12475 |
| **Pool 2** | BVDV2_324F | TGG ACA CAG CCT GAT AGG G | 316 |
|  | BVDV2_1065R | CTT CTT CAC CTG GTA CTT AAC C | 1033 |
|  | BVDV2_1707F | GGA GCA CGA TTG CGG CAA C | 1696 |
|  | BVDV2_2546R | CTC TCT GGY CCC AAT AAC C | 2517 |
|  | BVDV2_3067F | CCT ATC AAG AAG TGT AAG TGG TG | 3056 |
|  | BVDV2_4064R | AAG CAT GGG TCC ATW GCA C | 3813 |
|  | BVDV2_5307F | TGT GAA GGG ARA GAT TGG AAT G | 5075 |
|  | BVDV2_6489R | GGA TGC TTC AAA AAT TGG TAG CC | 5865 |
|  | BVDV2_7727F | CCG CAG TGA AGA ACA TCA TGG | 7125 |
|  | BVDV2_8555R | AGR TCA ACT GCT GCT TGC TTG | 7937 |
|  | BVDV2_9713F | CTR GTA AAG AAG CAC ACA GGG | 9113 |
|  | BVDV2_10481R | GCT TCC CTA TTG AGA AAG GTC C | 9858 |
|  | BVDV2_11599F | CCA AGA GTA ATC CAG TAC CC | 10997 |
|  | BVDV2_12387R | CTA CGG CCT TCT CAT AAG CTG | 11763 |
| **Pool 3** | BVDV2_394F | CAC ATG GAG TTG WTT TCA AAT GAA C | 383 |
|  | BVDV2_1065R | CTT CTT CAC CTG GTA CTT AAC C | 1033 |
|  | BVDV2_2433F | AGG GCT ATT GTG GCT GAT GC | 2422 |
|  | BVDV2_3206R | ACA CCA CCC CTA TCG CAA G | 3177 |
|  | BVDV2_3705F | GAA ATG GAT TAT ACT RGT GTA CCA C | 3694 |
|  | BVDV2_4727R | GTC AAA AAG TCT GCC CAC ATG G | 4473 |
|  | BVDV2_6282F | CAT CAA GAC CGA CTC AGG G | 5680 |
|  | BVDV2_7037R | GCT GGG GTT GCT GTC ATT G | 6417 |
|  | BVDV2_8391F | GGA GGA GAT CAT CAG GTA TGG | 7789 |
|  | BVDV2_9148R | CCC ACC CCT TAT AGT ACA CC | 8527 |
|  | BVDV2_10359F | CAA CCC AAG AGA GAT CAG GG | 9757 |
|  | BVDV2_11090R | GAT TTR GTT CTT AGG TTC CCT TC | 10466 |
|  | BVDV2_12271F | AAC ACC AGT AGT TAC ATG GCA G | 11672 |
|  | BVDV2_12890R | CTG YCA TCA GCA TCA SCC TC | 12268 |
