# Supplementary material for: Cattle connection: molecular epidemiology of BVDV outbreaks via rapid nanopore whole-genome sequencing of clinical samples
Source: BMC Vet Res. 2021 Jul 12;17:242. doi: 10.1186/s12917-021-02945-3 (PMC8272987; doi:10.1186/s12917-021-02945-3)
Supplement: Supplementary file 3 — Additional file 3. Additional sequencing data and GenBank accession number. [file 12917_2021_2945_MOESM3_ESM.docx]

**Cattle Connection: Molecular Epidemiology of BVDV Outbreaks via Rapid Nanopore Whole-Genome Sequencing of Clinical Samples**

Jacqueline King, Anne Pohlmann, Kamila Dziadek, Martin Beer and Kerstin Wernike

**Legend:**

**Additional file 3:** Additional sequencing data including mean genome coverage, consensus length, GenBank accession number and top two NCBI Blast results (date: 05.01.2021) with identity levels in percent

**Additional file 3**

Additional sequencing data including mean genome coverage, consensus length, GenBank accession number and top two NCBI Blast results (date: 05.01.2021) with identity levels in percent

|  | **Sample ID** | **Genome**  **Coverage** | **Length** | **Accession No.** | **Closest Relative**  **Accession No.** | **Identity %** | **Closest Relative**  **Accession No.** | **Identity %** |
| --- | --- | --- | --- | --- | --- | --- | --- | --- |
| 1 | 2016BVD01435 | 96% | 12,002 | MW528224 | KJ620017.1 | 92.31% | KJ689448.1 | 92.03% |
| 2 | 2016BVD01436 | 96% | 11,953 | MW528225 | KJ620017.1 | 91.87% | KJ689448.1 | 91.54% |
| 3 | 2018BVD01695 | 100% | 12,065 | MW528226 | KX987157.1 | 85.76% | LT837585.1 | 85.32% |
| 4 | 2018BVD06212 | 100% | 12,132 | MW528227 | KT951840.1 | 94.14% | KC757383.1 | 94.06% |
| 5 | 2018BVD06214 | 100% | 12,129 | MW528228 | KT951840.1 | 94.14% | KC757383.1 | 94.06% |
| 6 | 2019BVD04871 | 100% | 12,129 | MW528229 | KT951840.1 | 94.08% | KC757383.1 | 94.00% |
| 7 | 2019BVD04882 | 100% | 12,129 | MW528230 | KT951840.1 | 94.04% | KC757383.1 | 93.96% |
| 8 | 2019BVD04888 | 100% | 12,129 | MW528231 | KT951840.1 | 93.90% | KC757383.1 | 93.91% |
| 9 | 2019BVD04889 | 100% | 12,130 | MW528232 | KT951840.1 | 94.02% | KC757383.1 | 93.95% |
| 10 | 2017BVD04597 | 100% | 12,241 | MW528233 | KJ620017.1 | 93.78% | KJ689448.1 | 93.40% |
| 11 | D66/11-28 | 100% | 12,181 | MW528234 | KT832818.1 | 96.42% | HG426484.1 | 90.32% |
| 12 | TV02/13, T136 | 100% | 12,286 | MW528235 | HG426490.1 | 99.90% | HG426484.1 | 99.89% |
